# Pulmonary function in patients with adolescent idiopathic scoliosis: an explorative study of a wearable smart shirt as a measurement instrument

**DOI:** 10.1007/s43390-024-00938-4

**Published:** 2024-07-31

**Authors:** N. Te Hennepe, V. L. J. M. Steegh, M. H. Pouw, J. Roukema, M. De Kleuver, M. L. Van Hooff

**Affiliations:** 1 Department of Orthopedic Surgery, Radboud University Medical Center, Nijmegen, The Netherlands; 2 Department of Pediatrics, Division of Respiratory Medicine, Amalia Children’s Hospital, Radboud University Medical Centre, Nijmegen, The Netherlands; 3 Department of Orthopedic Research, Radboud University Medical Center, Nijmegen, The Netherlands; 4 Department of Research, Sint Maartenskliniek, Nijmegen, The Netherlands

**Keywords:** Adolescent idiopathic scoliosis, Pulmonary function, Respiratory function, Wearable technology

## Abstract

**Purpose:**

Adolescent idiopathic scoliosis (AIS) presents various challenges, including respiratory symptoms that impact pulmonary function. This study aims to explore the feasibility of using a smart shirt for continuous monitoring of lung volumes and heart rate during routine activities in AIS patients.

**Methods:**

A single-center exploratory feasibility study was conducted with AIS patients aged 16–22 years with a thoracic curvature of ≥ 30 degrees and absence of respiratory comorbidities. A smart shirt was utilized to continuously monitor cardiopulmonary parameters during mild exercise, which included a standardized walking route with the ascent of multiple stairs.

**Results:**

Five participants completed the study. Baseline spirometry measurements showed a range of values for forced vital capacity (FVC), forced expiratory volume in 1 s (FEV_1_), and FEV_1_/FVC ratio. During mild exercise, participants exhibited variability in tidal volume, heart rate, breathing rate, and minute ventilation, with increases observed during stair climbing. Breathlessness levels also varied throughout the activity but did not correlate with the measured lung volumes. Overall, the use of the smart shirt for assessing pulmonary function in AIS patients was deemed feasible and well tolerated by participants during the test activities.

**Conclusion:**

The study confirms the feasibility of using a smart shirt for continuous measurement of cardiopulmonary parameters in AIS patients during daily activities. Incongruities between spirometry results and perceived dyspnea exists, which questions the nature of the perceived dyspnea. Further research is needed to validate these findings and explore the impact of AIS characteristics on measurement accuracy.

## Introduction

Adolescent idiopathic scoliosis (AIS) is associated with various symptoms, including back pain, diminished self-image, physical limitations, and cardiopulmonary compromise [1, 2]. The impact of AIS on pulmonary function has been identified as a significant area of concern [3]. As the scoliosis progresses and the curvature of the spine and trunk deformity become more pronounced, individuals often experience respiratory symptoms such as breathlessness during routine activities. Respiratory problems and a reduced ability to tolerate exercise are commonly observed. Decreased lung volumes correlate with the severity of the scoliosis [4]. Factors beyond the magnitude of the coronal curvature, such as reduced thoracic kyphosis, vertebral rotation, general muscle dysfunction, diminished respiratory muscle strength, and particularly decreased chest wall compliance, can all potentially influence pulmonary function [1, 5, 6].

Recent studies have also indicated that spinal-thoracic deformities can lead to malformations in the bronchial airways, resulting in increased airway resistance [7]. Limitations in ventilation can lead to disability, which in turn may contribute to the avoidance of activities and exercise among AIS patients [8].

Both physicians and patients have recognized the impact of pulmonary problems and exercise intolerance. In 2017, a standardized set of outcomes for AIS was developed using the Delphi method [3]. Initially encompassing “pulmonary function” as a pertinent outcome domain, it was subsequently redefined as “pulmonary fatigue” to better encapsulate patients’ perspectives on pulmonary well-being. An underlying theoretical framework for this domain is imperative for future accurate assessment. The significance of pulmonary function in AIS has been acknowledged not only by physicians but has also been explored in patients with spinal deformities. Many patients encounter pulmonary symptoms, such as breathlessness during exertion and reduced exercise tolerance, which often curtail their daily activities [9].

How to measure pulmonary function in AIS remains unclear. It is commonly assessed using spirometry, involving a single-timed, forced measurement that does not accurately represent respiratory function during typical daily activities or exercise [10]. Multiple studies have demonstrated that lung volumes measured using CT scans correlate significantly with those measured by standard pulmonary function tests [11–13]. These findings suggest that externally measured lung volumes can be directly related to measured airflow. Recently, a novel ‘smart shirt’ [14] has been developed to continuously monitor cardiopulmonary function by measuring chest circumference at two levels. By employing this shirt, it may potentially also be possible to continuously monitor the lung volume of AIS patients, offering insights into pulmonary capacities during everyday activities and potentially shedding light on the relation between fatigue and pulmonary function.

Hence, the aim of the current study is to explore the feasibility of wearing a smart shirt to gauge lung volumes as an outcome measure during routine activities in patients with AIS.

## Methods

### Study design and patients

This study is a single-center explorative pilot feasibility study to investigate the feasibility of using a ‘smart shirt’ as a novel measurement instrument for assessing pulmonary function in a population of adolescents and young adults diagnosed with adolescent idiopathic scoliosis (AIS). Patients were included when they met the following criteria: age between 16 and 35 years, diagnosed with AIS with a thoracic coronal curvature of ≥ 30 degrees, ability to walk for 30 min and willingness to participate. Exclusion criteria were (a history of) smoking, having any respiratory comorbidity, active use of pulmonary medication, previous spinal surgery, or current brace treatment.

### Smart shirt description

The smart shirt (Hexoskin® Pro Shirt) is an novel wearable technology designed to monitor and analyze various physiological parameters real-time. To analyze the cardiorespiratory function, the shirt continuously monitors chest circumference at two specific levels: upper chest (at the lower end of the sternum) and the abdomen (approximately at the level of the umbilicus). Depending on the sex, height and age of the patient, it calculates the lung volumes. Furthermore, it has three electrocardiographic leads to continuously produce a 1-lead electrocardiogram (ECG, 256 Hz) and a 3-axis accelerometer. The shirt assesses breathing rate (2–60/min), heart rate (30–220 beats/min), minute ventilation (L/min) and tidal volumes (mL).

### Measurement procedure

Participants were fitted with the appropriate size of the smart shirt, ensuring good fit. Next, participants underwent pulmonary function testing (PFT) by spirometry using a calibrated spirometer (Vyntus ONE (2020), Vyaire Medical GmbH). PFTs were conducted by a trained pulmonary function technician according to ATS/ERS criteria [15, 16]. This was performed to assess the participants’ pulmonary status. The smart shirt was not calibrated against the spirometric measurements. Following spirometry, participants embarked on a 2.8-km walk along a standardized route, including the ascent of four flights of stairs at the end of the route. Smart shirt measurements began after 2–3 min of walking (distance from spirometer to starting point). Participants were instructed to walk and climb the stairs at a normal walking speed. First the ascent of two flights of stairs, followed by 2–3 min walking, then one flight of stairs, followed by 2–3 min walking and lastly again one flight of stairs which was concluded with another 2–3 min walking.

The level of breathlessness, as experienced by the participant, was evaluated both prior to and at various standardized points during the activity, i.e., after 10 min, after 20 min, and at the end of climbing the stairs, as well at conclusion of the walk. Breathlessness was assessed using a Dutch version of the Modified Borg dyspnea scale (*m*Borg scale). The *m*Borg scale is a subjective rating scale used to assess a person’s level of breathlessness or dyspnea during physical activity or exercise. It is a numerical scale ranging from 0 to 10, where 0 represents “no breathlessness at all” or “no effort,” and 10 indicates “maximum breathlessness” or “maximal effort” [17, 18]. The walking test was conducted partly outdoors and partly indoors. To assess the comfort of the shirt, participants were asked to score the comfortability after the walk concluded, ranging from 0 (“very uncomfortable”) to 10 (“very comfortable”).

### Description of measured parameters [19, 20]

The forced vital capacity (FVC), forced expiratory volume in 1 s (FEV_1_), and the FEV_1_/FVC ratio were assessed with spirometry at baseline. The smart shirt continuously measured tidal volume, breathing rate, heart rate, and minute ventilation.

#### FVC

FVC refers to the maximum amount of air that can be forcefully exhaled following a single, deep inhalation.

#### FEV_1_

FEV_1_ refers to the volume of air that can be forcibly exhaled during the initial second after taking a deep breath.

##### FEV_1_/FVC ratio

FEV_1_/FVC represents the ratio of the air volume exhaled within the first second of a forced exhalation effort to the total air volume exhaled during the FVC test. This parameter is used to identify two classic patterns in spirometry results: obstructive and restrictive patterns.

##### Tidal volume

Tidal volume refers to the amount of air a person inhales or exhales during a normal, quiet breath, typically in a single breath cycle. It represents the volume of air that moves in and out of the lungs with each breath when a person is at rest and not exerting themselves.

##### Minute ventilation

Minute ventilation describes the total volume of air a person inhales and exhales in one minute. It is calculated by multiplying the tidal volume by the respiratory rate (the number of breaths taken per minute).

### Data analysis

Since this study was exploratory and represents a pilot case series, only descriptive statistics were used. Characteristics were described per participant, including age, sex, BMI (kg/m^2^), Cobb angle (thoracic curve), and Lenke type. The baseline spirometry values are presented per participant. Although ‘normal’ FVC and FEV_1_ are dependent on multiple factors (e.g., age, sex, length, and ethnicity), the lower level of normal was roughly 80%, meaning that a measured FVC or FEV_1_ of at least 80% of the predicted value was considered as normal [21]. The FEV_1_/FVC ratio was described as ‘normal’ when it was within 10.4% of the predicted ratio [21]. The data acquired from smart shirt measurements were described over time. The raw data were downloaded from an online database and exported in Excel for descriptive analysis. No further statistical analyses were performed. The amount of breathlessness was described across the recorded timeframe.

### Ethical considerations

The study was conducted in compliance with the regulations and guidelines set forth by our institutional review board (IRB). The study protocol was reviewed and approved by the regional medical ethical review board (METC-Oost Nederland; 2022-13282). Prior to participation, all study participants were provided with information about the study’s objectives, procedures, and potential risks and benefits. Informed consent was obtained from all participants.

## Results

### Participant characteristics (Table 1)

**Table 1:** Characteristics of included patients

Patient	1	2	3	4	5
**Characteristics participants**					
Age (y), sex	20, F	16, F	20, M	16, M	22, F
Length (cm)	170	148	180	170	169
Weight (kg)	61	52	60	64	63
BMI (kg/m^2^)	21.2	23.7	18.5	22.1	22.1
Thoracic coronal Cobb angle (°)	45	63	63	35	65
Lenke Classification	1A	3C	1A	1A	2A
**Pulmonary function parameters (spirometry)**					
FVC (L)	3.74	3.09	3.75	3.77	3.52
FVC (%)*	89	104	66	83	85
FEV_1_(L)	3.24	2.7	2.78	3.37	3.12
FEV_1_ (%)*	89	101	58	86	87
FEV_1_/FVC(%)	86.8	87.4	72.4	88	88.5
FEV_1_/FVC (%)*	99	97	85	102	102

*F* female; *M* male, * percentage of the predicted value (based on age, height and weight), *L* absolute values, in liters, *FVC* forced vital capacity, *FEV*_*1*_ forced expiratory volume in 1 s

A total of six participants were initially enrolled in the study. Data from the final participant were lost due to technical issues. The saved file could not be located by the software, rendering it impossible to upload. Consequently, the data from this participant were excluded from the analysis. As it was a single time measurement, no patients were lost in follow-up. Therefore, in total, five patients were included. The ages of the remaining five participants ranged from 16 to 22 years. Among these participants, there were three females and two males. The length of the participants varied from 148 to 180 cm, and the mean body mass index (BMI) ranged from 18.5 to 23.7 kg/m^2^. The Cobb angle ranged from 35 to 65 degrees. Three participants were classified as Lenke 1A, one participant as Lenke 2A, and one participant as Lenke 3C.

### Baseline pulmonary function test (Table 1)

The FVC ranged from 3.09 to 3.75 L, and the predicted values from 66 to 104% (based on age, height, and gender). The FEV_1_ ranged from 2.70 to 3.37 L, and the predicted values from 58 to 101%. The FEV_1_/FVC ratio ranged from 72.4 to 87.4%, and the predicted values from 85 to 102%.

### Descriptives of walking route (Table 2)

**Table 2:** Descriptives of the walking route (2.8 km)

Patient	1	2	3	4	5
Total duration (min)	32:58	35:20	32:58	32:01	30:54
Start first flight of stairs (min)	23:50	27:28	24:16	23:51	23:33
Start second flight of stairs (min)	27:07	30:03	27:41	27:10	25:49
Start third flight of stairs (min)	29:51	32:17	30:11	29:41	28:03
Mean walking speed (km/h)	5.1	4.8	5.1	5.3	5.4

The total duration, instances of climbing the stairs and average walking speed is shown in Table 2. The average walking speed varied from 4.8 to 5.4 km/h. Each participant covered a walking route of 2.8 km, resulting in a total walking duration of 31–35 min.

### Smart shirt measurements

#### *Tidal volume (TV; *Fig. 1*)*

**Fig. 1:**
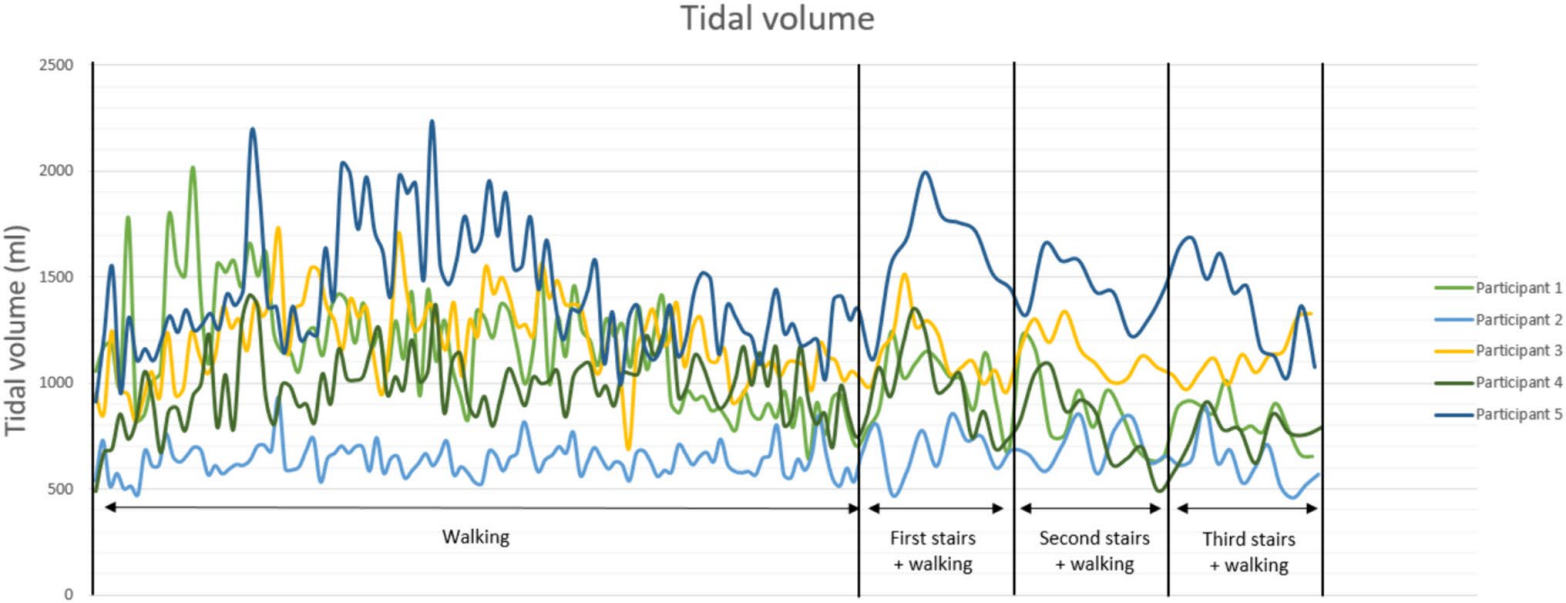
Graph demonstrating the tidal volume per distance, as generated by the smart shirt, for five patients with AIS

The TV measurements collected using the smart shirt revealed large variability among participants. It ranged from approximately 500 mL to 2000 mL. Participant 1, 3, 4 and 5 showed an increase in tidal volumes after 1 to 3 min of walking and a significant rise during walking the stairs. Participant two showed a minimal increase during walking and a modest increase during climbing the stairs.

### *Heart rate* (*HR*; Fig. 2)

**Fig. 2:**
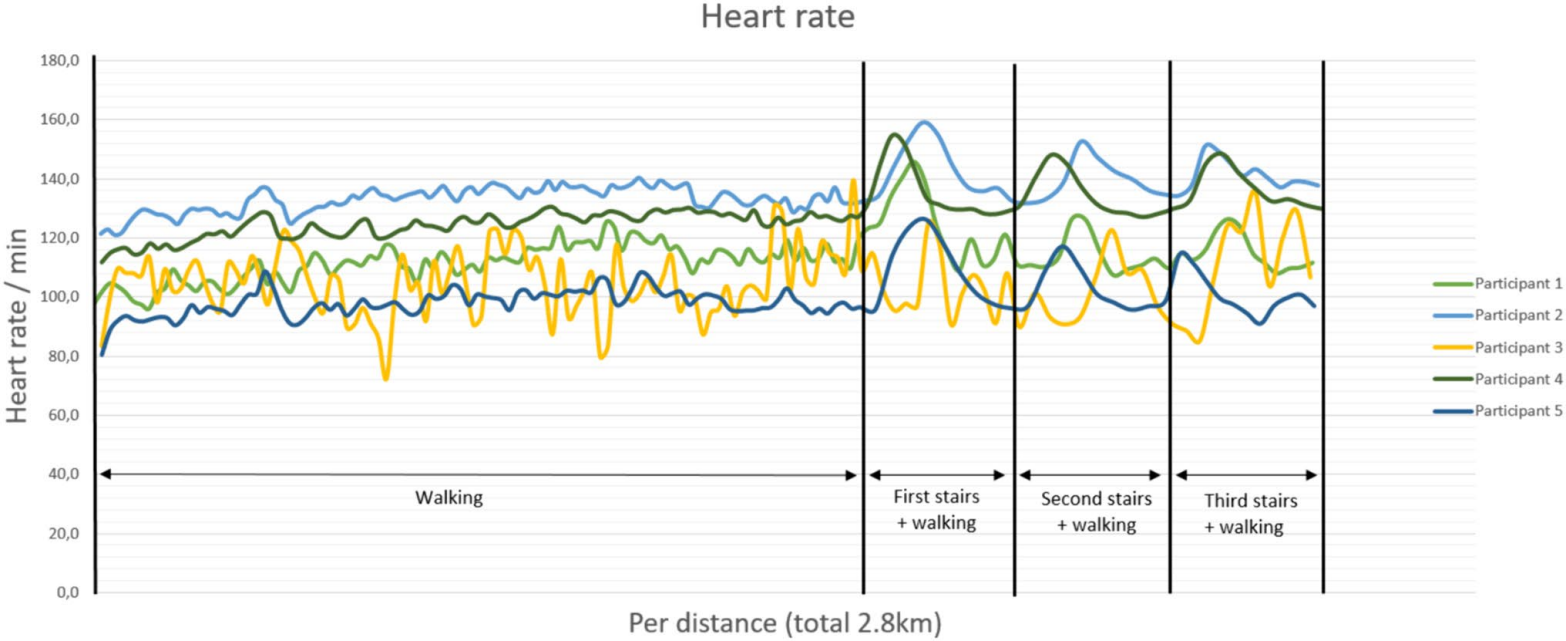
Graph demonstrating the heart rate per distance, as generated by the smart shirt, for five patients with AIS

The HR measurements also varied greatly between participants, from approximately 80–120 beats per minute. The heart rate showed a slight increase during walking, and a greater increase during climbing the stairs.

### *Breathing rate (BR; *Fig. 3*)*

**Fig. 3:**
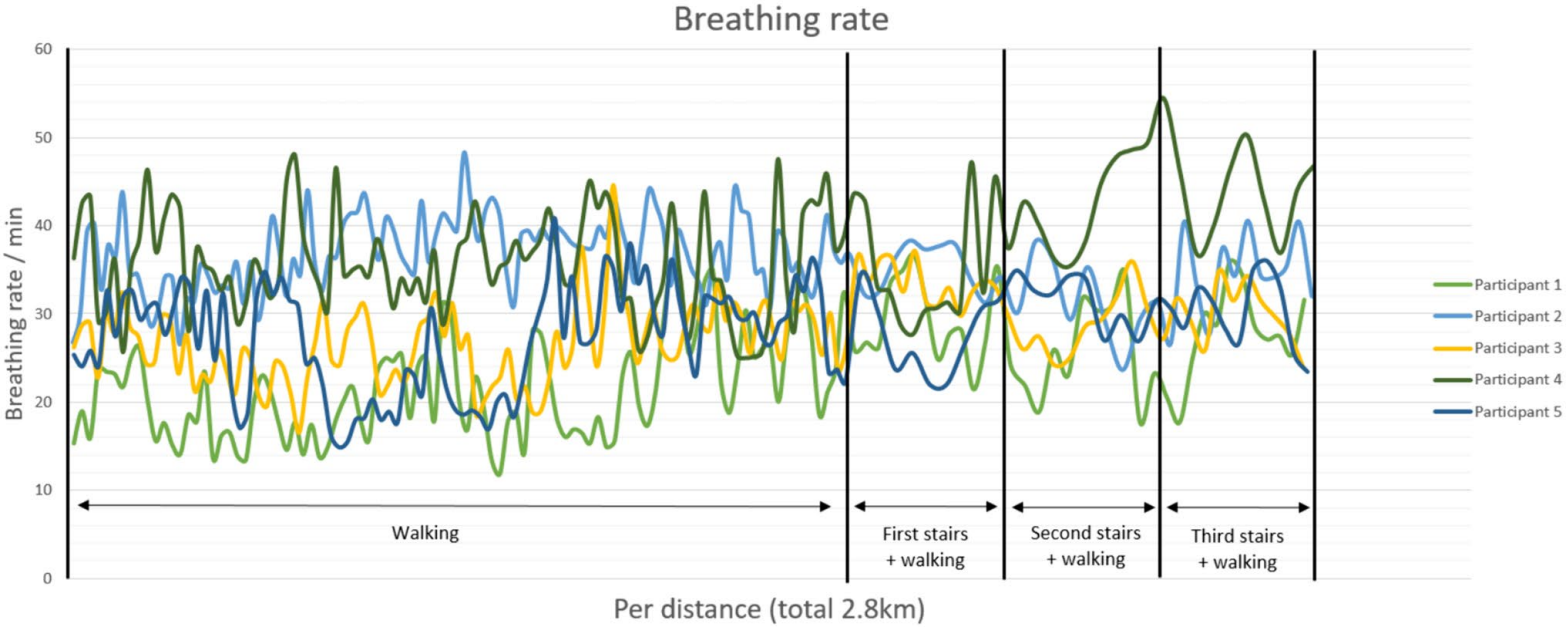
Graph demonstrating the breathing rate per distance, as generated by the smart shirt, for five patients with AIS

The BR ranged from approximately 15–40 per minute. Over time, no evident increase is seen during walking, and only participant four showed an increase during climbing the stairs. Participant four showed a significant increase in breathing rate during climbing the stairs.

### *Minute ventilation (MV; *Fig. 4*)*

**Figure 4:**
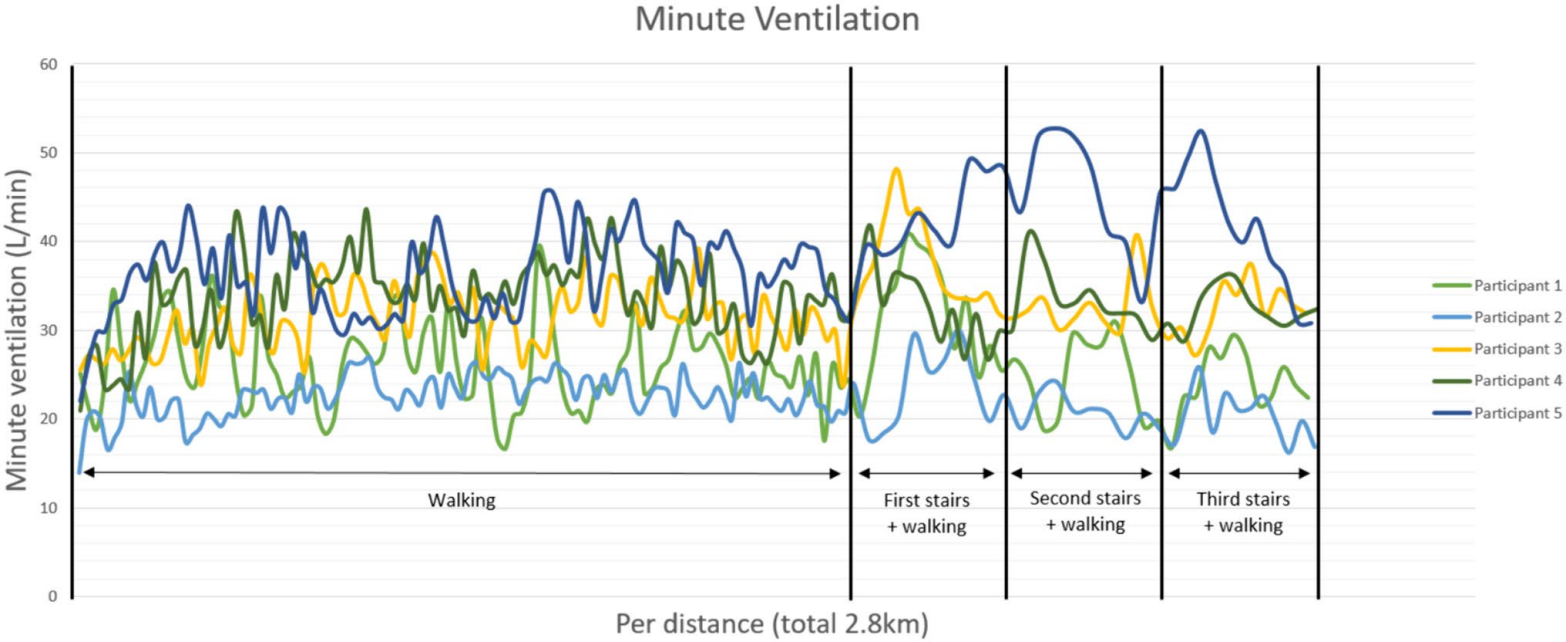
Graph demonstrating the minute ventilation per distance, as generated by the smart shirt, for five patients with AIS

The smart shirt facilitated the calculation of MV by multiplying TV and BR. Due to the increase in TV and a slight increase in BR, the MV increased in every participant during walking and increased further during climbing the stairs. Participant five showed a substantial increase during climbing the stairs.

### Breathlessness (mBorg scale; Table 3)

**Table 3:** Breathlessness (modified Borg dyspnea scale) and comfortability (0–10)

Patient	1	2	3	4	5
Start	3	1	1	0	2
After 10 min	7	2	1	2	2
After 20 min	5	5	2	3	2
After first flight of stairs	8	8	4	5	3
At finish	N/A	7	2	6	2
Comfort score	9	8	8	6	8

The initial level of breathlessness ranged from 0 to 3 (out of 10) and showed an increase after 10 min which ranged from 1 to 7. At the 20-min mark, the level of breathlessness decreased for participant one from 7 to 5, while for participant two to five, it increased or stayed the same (range 2–5). After climbing the first flight of stairs, participants reported a notable escalation in breathlessness, ranging from 3 to 8. As the walk concluded, the level of breathlessness ranged from 2 to 7. The amount of breathlessness at the finish from participant one was lost.

### Feasibility and compliance (Table 3)

The utilization of the smart shirt as a tool for assessing pulmonary function in AIS participants was determined to be both “feasible” and “well tolerated”. Nearly all the participants reported a high level of comfort with the shirt, giving it scores of 9, 8, 8, 6, and 8 out of 10.

## Discussion

This study has shown that measurement of cardiopulmonary parameters in adolescent idiopathic scoliosis (AIS) patients with a smart shirt is feasible. The smart shirt has been clinically validated in various studies, predominantly in a healthy population [22–28], with additional assessments extending to patients with COPD [29]. Tidal volume [22, 26, 28, 29], heart rate [22–26], breathing rate [22, 23, 25–27, 29] and minute ventilation [22, 25, 26, 29] seem to be accurately measured by the smart shirt. The measurements in these studies encompassed diverse physiological conditions, including running [27], periods of rest, submaximal exercise and maximal exercise [26], routine activities of daily living [22, 28, 29], running [27], (high-intensity) cycling [24, 25] and walking [22, 23]. The studies validating heart rate [22–26] consistently reported high interclass correlation coefficients (ICCs), with Pearson’s correlation coefficients across different exercise loads all exceeding 0.90. These measurements were conducted using widely validated clinical tools, including 3 or 12 lead ECG measurements and metabolic cart systems. Similarly, the studies validating breathing rate [22, 23, 25–27] also demonstrated high ICCs, with values often exceeding 0.96 and consistently above 0.84. These assessments were performed using breath-by-breath measurement systems or metabolic cart systems. One study was an abstract, so detailed information was not found [29]. For tidal volume assessments [22, 26, 28], good accuracy was reported [22], with ICCs ranging from 0.69 to 0.82 when compared with breath-by-breath measurement systems at different exercise loads [26], and a mean bias of 0.6% compared to a spirometer [28]. Finally, studies evaluating minute ventilation [22, 25, 26] reported good correlations, with ICCs greater than 0.81 [26], ranging from 0.69 to 0.84 [25], or described as very comparable [22]. These measurements were validated using widely used breath-by-breath flow sensors. Beyond tidal volumes, heart- and breathing rate and minute ventilation, prior studies regarding the smart shirt have not addressed other pulmonary parameters.

The findings of this feasibility study offer insights into the potential applications of wearable technology in monitoring respiratory parameters among individuals with AIS. The utilization of this smart shirt as a measurement instrument for pulmonary function assessment was found to be well tolerated and accepted by AIS patients during the test activities. Moreover, it offers preliminary insights into the real-time pulmonary performance of AIS patients, contributing to a deeper understanding of the theory underlying their pulmonary complaints.

Pulmonary complaints, as observed in patients with AIS, have conventionally been believed to be the result of a restrictive nature. This implies a limitation in the total lung air volume attributed to deformities in the thoracic cage, thoracic spine shortening, diminished chest wall compliance, decreased respiratory muscle effectiveness and increased stiffness [30, 31]. Notably, an inverse relationship appears to exist between the Cobb angle and diminished lung volumes, extending even to smaller curvatures [4]. The potential involvement of respiratory muscle dysfunction is mentioned as well [6, 32]. Contrary to conventional understanding, recent investigations propose an obstructive pattern originating from a right-sided bronchial constriction [33, 34]. This is due to intrusion of the spine into the chest resulting from the endothoracic hump induced by the deformity and is seen more in patients with loss of kyphosis. Furthermore, emerging evidence suggests a potential cardiac etiology for the perceived breathlessness, attributed to compression of the pulmonary artery leading to (mild) pulmonary hypertension [35, 36].

In a healthy individual, during mild to moderate exercise, the tidal volume and breathing rate typically increase which can result in a 12-fold increase of minute ventilation [37, 38]. In the majority of the study participants, tidal volumes exhibited an initial increase ranging from a factor of two to three (participant 1, 3, 4, and 5; Fig. 1), followed by a gradual decline over time, only to re-elevate during stair climbing. One participant (P2) displayed marginal increase in tidal volume. The overall breathing rate remained relatively constant, demonstrating only modest increases, except during stair ascent. While the heart rate exhibited minimal increments over time, a considerable rise was observed during stair climbing. The minute ventilation, calculated as the product of respiratory rate and tidal volume, displayed an overall increase, reached a plateau, and experienced a significant surge during stair climbing. The maximal increase in minute ventilation was noted to be a factor of 2.5.

**Fig. 5:**
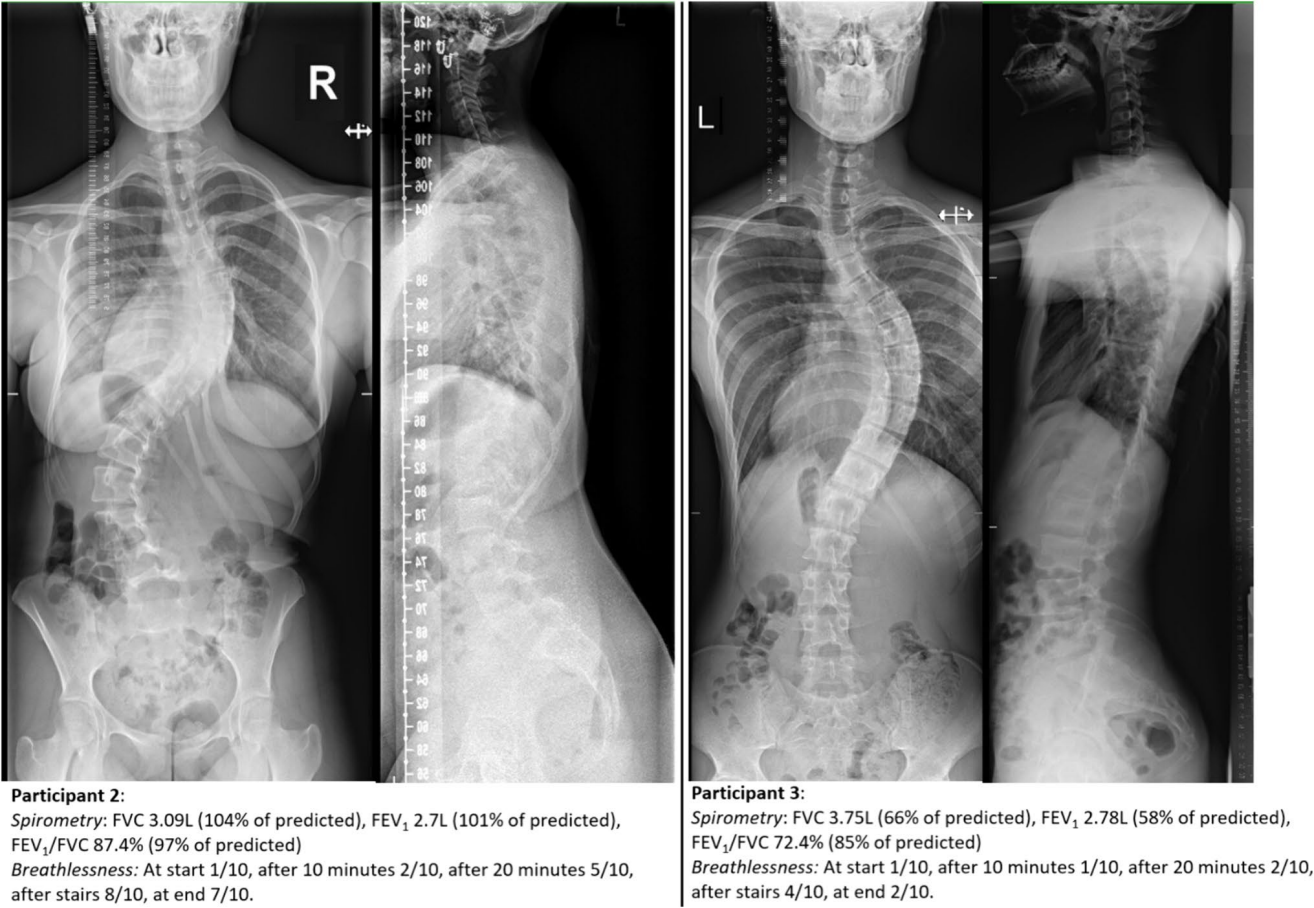
Posteroanterior (PA) and lateral full spine radiographs of participant two and three with similar curve magnitude and hypokyphosis, demonstrating markedly different spirometry and breathlessness measurements. Participant two has normal spirometry yet experiences significant breathlessness during exercise, whereas participant two demonstrates markedly reduced spirometry results but very little breathlessness during exercise

Surprisingly, the degree of perceived dyspnea as measured with the modified Borg scale did not demonstrate a discernible association with spirometry outcome or data obtained from the smart shirt. Notably, participants two and three exhibited comparable thoracic Cobb angles (63°; Table 1), yet their spirometric results varied greatly (Fig. 5). Participant two, despite displaying good spirometry values, reflected by forced vital capacity (FVC) and forced expiratory volume in one second (FEV_1_) exceeding 100% of predicted values (Table 2), reported substantial breathlessness (modified Borg dyspnea scale [mBorg], maximum score of 8/10; Table 3). Conversely, participant three exhibited decreased FVC (58%/predicted) and FEV_1_ (66%/predicted), yet reported a lower intensity of breathlessness (mBorg maximum score of 4/10). This incongruity suggests that spirometry in AIS patients may not offer the necessary insights into pulmonary symptoms as perceived by the patients.

Analysis of smart shirt data revealed that participant two, despite experiencing severe breathlessness, did not manifest an elevation in tidal volumes, only a marginal increase in heart rate (more pronounced during stair climbing), no conspicuous escalation in breathing rate, and a modest rise in minute ventilation. We assume that this caused by the inability of the participant to augment chest volume due to chest wall stiffness and reduced compliance or the potential presence of respiratory muscle dysfunction, as mentioned in prior literature [6, 32]. Additionally, cardiovascular factors should be considered a cause for dyspnea [35, 36]. In contrast, participant one, who similarly reported intense breathlessness, exhibited an initial increase in tidal volume, a slight elevation in heart rate (more pronounced during stair climbing), a modest rise in breathing rate, and a more pronounced increase in minute ventilation. She also had a normal spirometry but her Cobb angle was smaller (45 degrees). These measurements from spirometry and the smart shirt both have a poor correlation with self-reported pulmonary symptoms. This suggests a multifactorial nature of perceived dyspnea, or it could potentially be purely cardiac. These findings questions the clinical relevance of further prioritizing objectively measured lung volumes, such as those obtained through the use of a smart shirt.

### Strengths and limitations

To our knowledge, this is the first study in which lung volumes are continuously evaluated while performing activities. While this study provides valuable insights into the feasibility of employing the smart shirt to assess pulmonary function when performing daily activities in AIS patients, it is essential to acknowledge several limitations that should be considered in the interpretation of the findings. First, the smart shirt was not calibrated using spirometry. This means that the accuracy of the tidal volumes and minute ventilation cannot be established. Only the relative changes can be interpreted. Second, the sample size in this feasibility study was small. As a result, the findings may not be representative of the entire AIS population and may not account for potential variations in pulmonary function associated with different AIS characteristics, such as curve severity or patient age. However, the primary aim of this study was not to describe a representative AIS population but to outline a small case series to assess its feasibility. Third, wearable technology, such as the smart shirt, can be influenced by inherent technological limitations. For example, the fit of the shirt and signal interference have the potential to impact measurements accuracy. To prevent this, a glycerin-based ointment was applied to the sensors, as per the manufacturer’s recommendation. However, this may have contributed to the variable measurements in participant three compared to others. In future research endeavors, repeated measurements in the same patients would need to be performed to test the reproducibility of the measurements. Fourth, this feasibility study did not include a healthy control group without AIS and we cannot compare our data with the normal population. This would be interesting for a follow-up study.

## Conclusion

In conclusion, this feasibility study demonstrates the promise of a smart shirt as a well-tolerated tool for the continuous monitoring of cardiopulmonary parameters in adolescent idiopathic scoliosis (AIS) patients. It offers valuable insights into cardiopulmonary changes during mild exercise in this patient population. However, in this small exploratory study, we could not identify any relation between spirometry findings, smart shirt measurements or perceived dyspnea. To delve deeper into the alterations in lung volumes during activities in patients with AIS, further research is necessary. We recommend further evaluation of the smart shirt in AIS patients, as the chest mobility and, consequently, measurement accuracy may be affected by the thoracic deformity.

## Data Availability

The data that support the findings of this study are openly available in the Radboud Data Repository (RDR) at https://data.ru.nl/collections/ru/rumc/svpfais_t0000032a_dsc_538.
